# Imaging Evaluation of Pulmonary and Non-Ischaemic Cardiovascular Manifestations of COVID-19

**DOI:** 10.3390/diagnostics11071271

**Published:** 2021-07-15

**Authors:** Sebastiano Cicco, Antonio Vacca, Christel Cariddi, Rossella Carella, Gianluca Altamura, Antonio Giovanni Solimando, Gianfranco Lauletta, Fabrizio Pappagallo, Anna Cirulli, Assunta Stragapede, Nicola Susca, Salvatore Grasso, Roberto Ria

**Affiliations:** 1 Internal Medicine Unit “Guido Baccelli”, Department of Biomedical Sciences and Human Oncology (DIMO), University of Bari Aldo Moro, Azienda Ospedaliero-Universitaria Policlinico, Piazza G. Cesare 11, I-70124 Bari, Italy; corionfrondoso@libero.it (R.C.); antoniogiovannisolimando@gmail.com (A.G.S.); gianfranco.lauletta@uniba.it (G.L.); fabrizio.pappagallo@hotmail.it (F.P.); alisaciru@gmail.com (A.C.); assunta-s@live.it (A.S.); susnic2@gmail.com (N.S.); roberto.ria@uniba.it (R.R.); 2 Division of Internal Medicine, Department of Medicine, Building 8, University of Udine, I-33100 Udine, Italy; antonio.vacca94@gmail.com; 3 Anesthesiology and Intensive Care Unit, Department of Emergency and Organ Transplantation (DETO) Ospedale Policlinico, University of Bari Aldo Moro, Azienda Ospedaliero-Universitaria Policlinico, Piazza G. Cesare 11, I-70124 Bari, Italy; c.cariddi90@gmail.com (C.C.); gianlucaaltamura.ga@gmail.com (G.A.); salvatore.grasso@uniba.it (S.G.)

**Keywords:** cardiovascular involvement, cardiovascular magnetic resonance, coronavirus disease 2019, echocardiography, emergency, heart computerized tomography, intensive care, lung ultrasound, nuclear imaging, point-of-care ultrasonography

## Abstract

Coronavirus Disease 2019 (COVID-19) has been a pandemic challenge for the last year. Cardiovascular disease is the most described comorbidity in COVID-19 patients, and it is related to the disease severity and progression. COVID-19 induces direct damage on cardiovascular system, leading to arrhythmias and myocarditis, and indirect damage due to endothelial dysfunction and systemic inflammation with a high inflammatory burden. Indirect damage leads to myocarditis, coagulation abnormalities and venous thromboembolism, Takotsubo cardiomyopathy, Kawasaki-like disease and multisystem inflammatory syndrome in children. Imaging can support the management, assessment and prognostic evaluation of these patients. Ultrasound is the most reliable and easy to use in emergency setting and in the ICU as a first approach. The focused approach is useful in management of these patients due its ability to obtain quick and focused results. This tool is useful to evaluate cardiovascular disease and its interplay with lungs. However, a detailed echocardiography evaluation is necessary in a complete assessment of cardiovascular involvement. Computerized tomography is highly sensitive, but it might not always be available. Cardiovascular magnetic resonance and nuclear imaging may be helpful to evaluate COVID-19-related myocardial injury, but further studies are needed. This review deals with different modalities of imaging evaluation in the management of cardiovascular non-ischaemic manifestations of COVID-19, comparing their use in emergency and in intensive care.

## 1. Introduction

Coronavirus Disease 2019 (COVID-19) is a pandemic caused by SARS-CoV-2 and arose in 2019 in Wuhan, China [1]. This new virus belongs to the Coronavirus family that includes the Severe Acute Respiratory Syndrome (SARS-CoV) and Middle East Respiratory Syndrome (MERS-CoV) viruses. All those have a tropism for lung cells [2]. In particular, SARS-CoV-2 enters the host cell using angiotensin-converting enzyme II (ACE2) as the cell surface receptor [2]. Acute COVID-19 shows different clinical manifestations, ranging from asymptomatic or less symptomatic up to severe/fatal disease. Bilateral interstitial pneumonia and moderate to severe oxygen desaturation and hypoxia are the most common presentations in severe patients that may develop Respiratory Failure (RF) and Acute Respiratory Distress Syndrome (ARDS), requiring prompt admission to intensive care unit (ICU). Since a direct cardiac involvement [1] was observed, it has been hypothesized as a direct effect of SARS-SoV-2 on myocardium and heart vessels [2,3]. There are many reports about the role on COVID-19 in spurring a diffuse endothelial inflammation [2,4,5] as a result of virus tropism for Angiotensin Converting Enzyme (ACE) 2. This enzyme is also expressed on the surface of type II pneumocytes [6]. These cells are anatomically closed to the lung vasculature and are described to be hyperplasic in COVID-19 lungs samples [7,8,9]. Inflammatory cells are involved in small vessel congestion, so called “immunothrombosis” [10]. The consequent lung tissue ischaemia may cause endothelial cells’ hyperplasia. A thrombosis of large vessels may occur as well [11]. Other coronaviruses can directly infect endothelial cells and may induce an endothelial dysfunction as an important pathophysiological event [12,13,14,15,16]. Similarly, SARS-CoV-2 directly infects endothelial vascular cells and leads to cell damage and apoptosis [17]. The decreased antithrombotic activity of the normal endothelium is direct consequence [11,18]. All these mechanisms contributed to RF inducing alveolar damage, vessel wall swelling, hyaline thrombi, microhaemorrhage and diffuse capillaries thrombosis [19,20,21]. Severe COVID-19 is characterized by high levels of pro-inflammatory cytokines (IL-1, IL-6 and TNF-α) and ferritin [16,22], activating endothelial cells of lung blood vessels. The role of inflammation has been also related to prognosis in COVID-19 [23,24] and to cardiovascular involvement. Recent in vitro studies have related the myocardial involvement to a direct SARS-CoV-2 action on cardiomyocytes in the form of diffuse cardiovascular involvement [25,26]. Demises related to cardiovascular disease increased during COVID-19 pandemic in both swab negative [27] and positive patients [28]. However, COVID-19 patients with cardiac involvement have a high mortality rate [29,30].

COVID-19 presents severe lung involvement and hemodynamic alterations that therefore induce commonly cardiovascular complications showing a wide spectrum of severity [31]. Clinical studies and several evidences show that patients with severe COVID19, as critically ill patients, generally are affected by the “cytokine storm”, which plays a crucial role in the disease progression and severity, and in influencing the COVID-19–associated ARDS and multiorgan dysfunction syndrome [32,33]. Cardiac involvement may occur in critically ill patients, as a consequence of mechanical ventilation as well as a direct effect of the SARS-CoV2 virus [33,34,35]. However, different approach should be used in cardiovascular evaluation of patients in emergency and intensive care unit (ICU) setting.

This review focuses on images that have proved to be very helpful for the evaluation of cardiovascular involvement in the COVID-19 patients.

## 2. COVID-19 Cardiovascular Involvement

Cardiovascular disease is the most described comorbidity in COVID-19 patients, and it is related to the disease severity. It is likely related to lung failure and COVID-19 disease progression [29,36]. However, cardiovascular involvement may arise also in children, [37] indicating that the heart involvement should be described also in patients without cardiovascular risk factors. The myocardial involvement may be considered as a part of the COVID-19 disease. In many patients, cardiovascular symptoms (such as heart palpitations and chest tightness) may be initial clinical manifestation of COVID-19 [38,39,40]. COVID-19 can exacerbate a pre-existing cardiovascular disease as well as it can cause new cardiovascular illness, as shown in several studies [1,41]. Described mechanisms of myocardial injury include imbalance of oxygen supply–demand, direct viral myocardial invasion, inflammation, coronary plaque rupture with acute myocardial infarction, microvascular thrombosis and adrenergic stress. In COVID-19 patients, the myocardial injury ranges between 9.6% and 46.3% [42]. Myocardial injury can be detected in about 25% of hospitalized patients affected by COVID-19, and it is associated with an increased mortality risk [43]. It causes worse prognosis, higher mortality rate (RR 5.54, 95% CI 3.48–8.80) and more ICU admissions (RR 3.78, 95% CI 2.07–6.89) [42]. 

**Figure 1:**
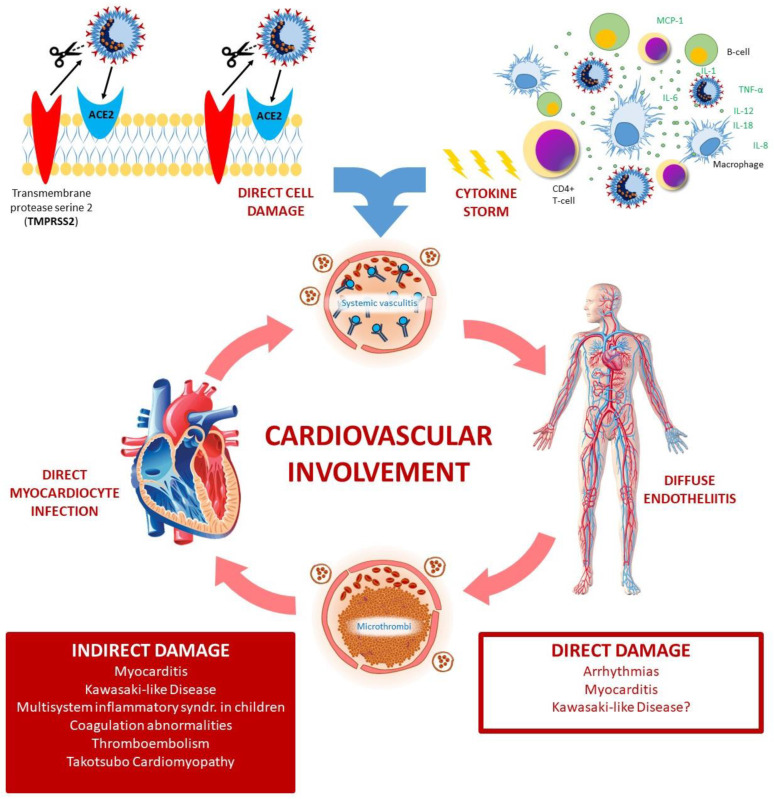
COVID-19 heavily affects patients’ cardiovascular system: it can induce direct damages, leading to arrythmias and myocarditis, and indirect damages, mediated by cytokine storm, systemic vasculitis and vascular thrombosis. The indirect damage contributes to the development of myocarditis and leads to Kawasaki-like Disease and Multisystem inflammatory syndrome, coagulation abnormalities and venous thromboembolism, and Takotsubo cardiomyopathy. ACE2: Angiotensin Converting Enzyme 2; IFN-γ = interferon-γ, MCP1 = monocyte chemoattractant protein 1, MIP1-α = macrophage inflammatory protein 1-α; ROS = Reactive Oxygen Species; TMPRSS2: Transmembrane protease, serine 2; TNF-α = tumour necrosis factor-α.

The high inflammatory burden due to cytokine release can significantly hit the patients’ cardiovascular systems [44,45,46,47]; it can induce arrhythmias, myocarditis, coagulation abnormalities with venous thromboembolism, Takotsubo cardiomyopathy, Kawasaki-like disease and multisystemic inflammatory syndrome, [48] leading directly or indirectly to cardiogenic shock [49]. SARS-CoV-2 uses the viral spike protein to bind ACE2 entering the host cell [50,51] (Figure 1). Host cell serine proteases TMPRSS2, cathepsin B, and cathepsin L primed the viral spike protein [52,53]. TMPRSS2 is present on lung cells expressing ACE2 and is mandatory for viral entry. Additionally Nicin et al. [54] showed that cardiac cells, (which include cardiomyocytes, pericytes, fibroblasts, endothelial cells and leukocytes from patients affected by heart failure and reduced ejection fraction or aortic stenosis), hugely express ACE2. SARS-CoV-2 cell entering could be facilitated by an underlying renin-angiotensin system (RAS)-related, pathophysiology of the cardiovascular disorders and chronic use of RAS inhibitors, which both induce an increase of the ACE2 levels [17,55]. Thus, the infection may have a direct impact on cardiovascular diseases [48]. However, ACE2 inhibitors reduce the levels of angiotensin II (AGII), a molecule that play also as a potent proinflammatory agent on lungs. Thus, AGII inhibition may contribute to reduce lung injury [56].

### 2.1. Myocarditis

SARS-CoV-2 infection affects the myocardium in the form of myocarditis (Figure 1). As mentioned before, ACE2 has a key role to initiate the COVID-19 infection, playing an important role in viral pathogenicity. The receptorial machinery may lead to a down-regulation of ACE2. This down-regulation produces toxic overaccumulation of angiotensin II and induces fulminant myocarditis, similar to acute respiratory distress syndrome [48]. 

Severe myocarditis with reduction of systolic function were reported as consequence COVID-19 [34,57]. Some patients died from virus-activated “cytokine storm syndrome” and/or fulminant myocarditis. An extremely robust cytokine storm given by interleukin-1 (IL-1), IL-2, IL-6, IL-8, TNF-α and MCP-1 is also the main pathophysiological mechanism described for fulminant myocarditis [16,41,58,59]. Furthermore, sporadic autopsy cases in fulminant myocarditis found an infiltration of interstitial mononuclear inflammatory cells in the myocardium [9,60,61].

Guo et al. reported an acute myocardial injury, defined as elevation of troponin T, in 28% of 187 hospitalized COVID-19 patients. Patients with high troponin T levels also presented higher inflammatory biomarkers, such as leukocytosis, lymphopenia, D-dimer, C-reactive protein and pro-calcitonin [62]. Thus, myocardial injury is mostly related with myocarditis due to viral infection. Ruan et al. [41] found that, on a sample of 150 patients confirmed COVID-19, the ones who died had higher levels of troponin, myoglobin, C-reactive protein, serum ferritin and IL-6; among 68 deaths, 7% were attributed to myocarditis with circulatory failure and 33% were cases in which myocarditis might contribute in the patient’s demise.

### 2.2. Coagulation Abnormalities and Venous Thromboembolism

COVID-19 patients are exposed to an elevated risk of arterial and venous thromboembolism (VTE) due to a state of endothelial dysfunction, vascular inflammation and hypercoagulability related to SARS-CoV-2 infection, and to its role in triggering innate immune response and inflammation, leading to immunothrombosis [10,63]. Gąsecka et al. analysed macrovascular and microvascular complications of these patients [64]. One third of patients hospitalized due to severe COVID-19 develop macrovascular thrombotic complications, including venous thromboembolism (VTE), which are associated with an increased risk of in-hospital mortality [64]. In about 31–40% of critically ill COVID-19 patients there is an increased risk of VTE [65,66]. In a recent study of 143 patients, deep vein thrombosis (DVT) was found with ultrasonography of the lower limbs in 46.1% of patients [67]. 

Regarding microvascular thrombosis, many severe patients develop COVID-19-associated coagulopathy, which differs from classic thrombotic microangiopathy and disseminated intravascular coagulation (DIC) [64]: the first autopsy series of four patients who died due to severe COVID-19 demonstrated the presence of diffuse microthrombosis and haemorrhage along with abundant megakaryocytes in all major organs, including the lungs, heart, kidneys and liver [20]. This pathological picture has been described as pauci-inflammatory thrombogenic vasculopathy because the gross pulmonary thromboembolism and parenchymal inflammation were absent. The abundance of megakaryocytes in the lungs at autopsy seems to colocalize with platelet-rich thrombi [64,68].

In these patients there are abnormal coagulation parameters, such as prothrombin time, activated partial thromboplastin time, fibrin degradation products and D-dimer. In particular, increased levels of fibrin degradation products (FDP) and D-dimer are closely associated with poor prognosis [63,69]. In a cohort of 1099 Chinese patients, 60% had severe illness and 46% had elevated D-dimer levels (>0.5 mg/L) [70]. Other studies confirmed that hospitalized patients with severe COVID-19 have abnormal coagulation parameters [69,71]. In about 31–40% of critically ill COVID-19 patients there is an increased risk of VTE [65,66].

Tang et al. [69] showed that FDP and D-dimer were significantly higher in COVID-19 non-survivors compared to survivors, and clinical criteria for disseminated intravascular coagulation (DIC) during the disease course were met in 71.4% of non-survivors. The DIC patients had high VTE rates, high fibrinogen levels, low antithrombin levels, elevated D-dimer levels and pulmonary congestion due to microvascular thrombosis and occlusion. Fibrin deposition in the pulmonary microvasculature contributed to ARDS in patients with concomitant diagnoses of DIC [72].

Hypercoagulability associate to a severe inflammatory state instead of acute DIC-like was described by Panigada et al. among 24 COVID-19 patients hospitalized in ICU [73]. They described increased C-reactive protein, normal or increased platelet count, increased fibrinogen, near-normal prothrombin time and activated partial thromboplastin time, and dramatically increased D-dimer. Factor VIII and von Willebrand factor increased, as well as protein C, while antithrombin marginally decreased [73]. 

Thus, current recommendations are to favour platelet inhibitors, which showed to be effective in improving hypoxemia and successful in weaning ventilator [74].

### 2.3. Takotsubo Cardiomyopathy

Takotsubo cardiomyopathy (TCM), also known as stress-induced cardiomyopathy, may be associated with the SARS-CoV-2, considering its increased incidence in the COVID-19 patients [75] (Figure 1). It is characterized by a transient reversible wall motion abnormality of the left ventricle (LV) due to an acute dysfunction in the setting of physical or emotional stress without significant obstructive coronary artery disease [76]. Apical TCM is the most common form (>80%) followed by the midventricular form [76]. It is typically associated with intense emotional or physical stress, and most commonly seen in women (>90%) [77]. There is very little literature on TCM pathophysiology, although acute stress condition leads to catecholamine surge and it has been suggested as pathophysiological mechanisms [78]. Severe systemic inflammation and cytokine storm could lead to acute stress and injury, pointed by elevated markers of myocardial injury such as C-reactive protein, pro-calcitonin, myoglobin, creatine kinase and N-terminal pro b-type natriuretic peptide (NT-proBNP) [79]. “Cytokine storm syndrome” in COVID-19 patients overlaps cytokine release syndrome [80]. The latter condition is accompanied by catecholamine surge [81], which can predispose to the TCM. 

Therefore, the association between TCM and COVID-19 may be explained by potential pathophysiological links between the two conditions. Though these direct connections are not fully understood, they may be attributable to three factors: the overactive immune response from cytokine storm, the sympathetic nervous system surge and the development of microvascular dysfunction noted in SARS-CoV-2 infection [75]. 

### 2.4. Kawasaki-Like Disease and Multisystem Inflammatory Syndrome

SARS-CoV-2 is associated with sharp increase in the incidence of Kawasaki-like Disease (KL) [82,83,84] (Figure 1). The Kawasaki disease is a systemic vasculitis with predilection for coronary arteries mostly affecting children <5 years of age. In Japan, North America and Europe it is the most common cause of acquired heart disease in childhood, even though it may be a self-limited febrile illness [85,86]. Furthermore, coronary artery aneurysms (CAAs) from the Kawasaki disease affect patients in adult life, especially those with missed diagnosis or delayed treatment. Those patients are at risk for coronary artery thrombosis, myocardial ischemia and infarction, and account for 5% of acute coronary syndromes [87]. The aetiology of Kawasaki disease is still unknown, but it is generally accepted that unknown virus can trigger it, because seasonal peaks of the disease parallels with common seasonal respiratory infections [88]. 

Skin biopsy from children presenting with chilblains showed endothelial cell damage, thrombosis and SARS-CoV-2 in endothelial cells [89]. 

COVID-19 was initially reported as affecting children only mildly, as showed in an epidemiologic survey performed by Chinese Centre for Disease Control and Prevention including 2135 SARS-CoV-2-exposed children as well as in other studies [90,91,92,93]. During the progression of the COVID-19 pandemic in Europe and USA, later cases series reported outbreaks of a severe multisystemic inflammatory syndrome (MIS) in SARS-CoV-2 exposed children. This new syndrome has been called “Multisystem Inflammatory Syndrome In Children” (MIS-C) by the World Health Organization (WHO) and the Centre for Disease Control (CDC) [94]. MIS-C shows similar features with staphylococcus aureus toxin-mediated toxic shock syndrome [28] and atypical Kawasaki disease [95,96]. It is a cytokine storm syndrome induced by SARS-CoV-2 with very high inflammation markers: C-reactive protein, pro-calcitonin, pro-inflammatory cytokine levels, mainly IL-6 and IL-10, soluble IL-2 receptor, ferritin, D-dimers lymphopenia and neutrophilia [97,98]. It frequently fulfils criteria for macrophage activation syndrome (MAS) of children with juvenile idiopathic arthritis/Still’s disease [99]. MIS-C is characterized by multi-organ dysfunction, high fever rash hypotension, gastrointestinal symptoms, conjunctivitis and mucosal changes, including also high frequency of myocarditis and shock from either acute myocardial dysfunction or systemic hyperinflammation. As a result, cardiac manifestations, including myocardial and coronary involvement, are frequent in children with COVID-19 related Kawasaki-like disease and/or MIS-C, and need to be carefully identified and monitored over time.

### 2.5. Arrhythmias and Heart Failure

Arrhythmias are another example of common cardiovascular involvement in patients with COVID-19. In the setting of the coronavirus infection, high prevalence of arrhythmias in patients with or without a prior cardiovascular disease might be partly attributable to hypoxia, metabolic disarray, neurohormonal or inflammatory stress. Myocardial injury is associated with impairment of cardiac function [100]. Atrial and ventricular arrhythmias are associate with fulminant myocarditis and cardiogenic shock [56,57,100]. In fact, a new onset of malignant tachyarrhythmias in the setting of troponin elevation should raise suspicion for an underlying myocarditis [101,102]. In a cohort of 137 COVID-19 patients, heart palpitations were described as part of the early symptomology in 7.3% of patients [56], which indicates the possibility of arrhythmia as a consequence of SARS-CoV-2 infection. Fatal outcomes of COVID-19 also have a significant association with myocardial injury and cardiac disfunction, leading to arrhythmias [103]. Wang et al. [103] reported that among 138 patients hospitalized with COVID-19, cardiac arrhythmia was revealed in 16.7% and contributed to the transfer to the ICU of 44% of the patients. Of note, respiratory symptoms may mask the manifestations related to arrhythmia in COVID-19 patients.

Heart failure (HF) can be induced by COVID-19 via different mechanisms, including virus-induced infiltration of inflammatory cells, pro-inflammatory cytokines that could cause necrosis and death of the myocardium, endothelial injury, acute respiratory distress syndrome (ARDS) and respiratory failure with severe hypoxia [104]. In a systematic review and meta-analysis Zuin et al. found that acute heart failure (HF) represents a frequent complication of COVID-19 associated with a higher risk of mortality in the short-term period [105].

Patients with HF are at increased risk for poor outcomes such as hospitalization, and death from COVID-19. There is a significant difference in mortality between patients with and without HF: those with HF show higher mortality rates [106].

## 3. Point-of-Care Ultrasonography

Healthcare system evaluates a huge number of patients with suspected COVID-19 infection during the pandemic. It is essential to use a rapid execution diagnostic method that is free of detrimental effects and contraindications, and repeatable, in order to better manage resources and optimise therapy for patients in the right care setting. Ultrasound meets all these requirements, but other methods such as CT, may be evaluated when establishing the diagnosis and the clinical management. Point-of-care ultrasonography (POCUS) has demonstrated to be a ubiquitous and quick way to detect pulmonary changes [107]. In particularly, the use of lung ultrasonography (LUS) in COVID-19 has received much attention. It has the advantage of an easy and quick assessment to identify and classify disease severity. Even if none of the LUS features is pathognomonic for COVID-19, there has been a great deal of evidence to support its clinical value. LUS may play a complementary role in the work-up of COVID-19. In fact, ultrasounds can be used to detect signs of pulmonary involvement, the disease progression and regression. However, due to the huge cardiovascular involvement in SARS-CoV-2 pneumonia, a comprehensive lung-cardiovascular assessment is needed, especially in the Emergency or ICU setting.

**Figure 2:**
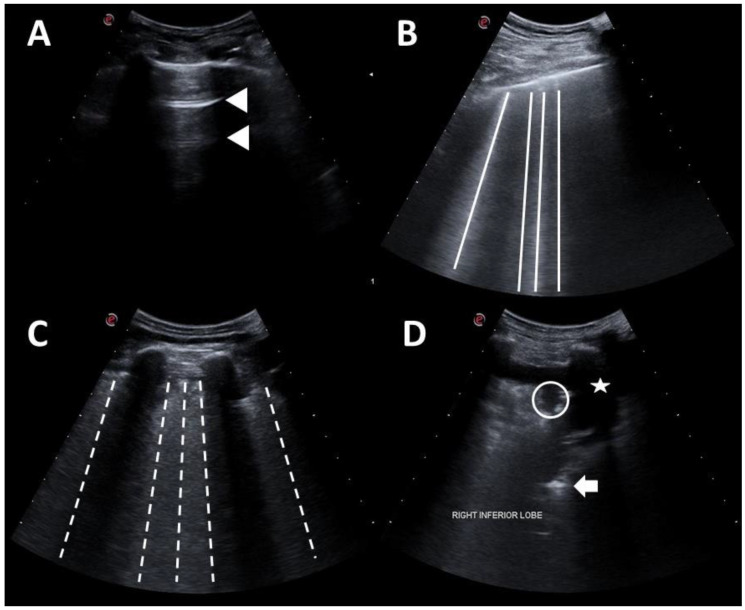
Lung ultrasonography grading score is part of BLUE protocol for evaluation of dyspnoea in emergency setting. (**A**) Score 0: normal pattern, A-lines or <3 B-lines; (**B**) score 1: moderate loss, ≥3 B-lines; (**C**) score 2: severe loss, coalescent B-lines; (**D**) score 3: complete loss, white lung and/or lung consolidations. Legend: pleural line is indicated by star; A-lines are indicated by triangles; B-lines are indicated by continue lines; coalescent B-lines are indicated by dashed lines; Consolidation is indicated by circles, aerial bronchogram is indicated by arrow, while pleural effusion is indicated by stars.

A possible useful algorithm for emergency evaluation is the A.B.C. algorithm. It is based on the three step process of cardiac resuscitation protocol: Airway (A), Breathing (B) and Circulation (C) [107]. The airway evaluation is based on the POCUS examination of no obstruction to patient airflow. Endotracheal intubation is the fundamental procedure for providing invasive mechanical ventilation, hence ultrasound represents a good method to confirm the correct endotracheal tube placement also in obese patients in absence of other device [108]. The direct sign of a correct placement of endotracheal tube is a hyperechoic shadow (comet sign), whereas an indirect sign is scanning for lung sliding during ventilation [109]. After the airways, dyspnoea assessment is mandatory. The BLUE protocol [110] is a standardized diagram for the rapid identification of the causes of dyspnoea, that can easily be used in imaging diagnosis of coronavirus disease (Figure 2).

The pleura is a hyperechoic structure. In a normal lung, it is detectable horizontal repetitions of the pleural line as a reverberation artifact, the A-lines. The association of the A profile with phlebothrombosis favours the diagnosis of pulmonary embolism (PE) [111]. The main LUS findings in interstitial syndromes are B-lines [112]. The presence of B-lines can refer to multiple lung disease. They are also present in patients with COVID pneumonia and become confluent with the disease progression [112]. Pattern B is indicative also for increased extravascular lung water. According to the M-BLUE protocol, twelve areas (bilateral superior BLUE point, M point, PLAPS point, diaphragm point) are assessed for each patient [110]. For this purpose, is used a semi-quantitative scoring system for the twelve regions (Figure 2). Therefore, a final score of 0 is normal whereas 36 would be the worst [110,113]. 

In the SARS-Cov-2 patients it has been also described the pleural line thickening showed as a thick hyperechoic pleural line and sub-pleural consolidations. The normal aerated lung tissue is replaced by tissue that mimics the aspect of other organs, for example liver; it is so called “the tissue-like sign” [114]. Consolidation should be confirmed by other signs. Lung pulse sign could help differentiation between lung consolidation and atelectasis: in the latter case lung results non-inflated, thus there is the heart beats’ transmission at the pleural line through the parenchyma [112,114,115]. Atelectasis is an important reversible cause for RF and can be diagnosed using ultrasound or chest radiography. Pleural effusions are rare findings in SARS-CoV-2 patients, though frequent in other critically ill patients [112].

SARS-CoV-2 can determine cardiac events related to the infection [30,116]. Profound hypoxemia underlying pneumonia together with tachycardia might result in chest pain and electrocardiographic changes suggestive of myocardial involvement [116]. All these events may induce an acute cardiovascular failure in COVID-19 patients.

Therefore, in patients with COVID-19 disease echocardiography should be performed on admission and should be repeated [107,117] periodically.

It has been explained how pulmonary thrombosis and arterial and venous thromboembolism affecting a sizeable proportion of patients in ICU, could cause DVT, PE, ischaemic stroke, myocardial infarction and systemic arterial embolism [118]. Zotzmann et al. [119] retrospectively evaluated all SARS-CoV2-associated ARDS patients admitted to ICU who underwent LUS and a CTPA. In addition, Wells score was calculated to evaluate PE probability. In 90% of patients, LUS found subpleural consolidation: PE-typical large subpleural consolidations sized ≥1 cm were detectable by LUS in 65% of patients and were significantly more frequent CTPA confirmed PE compared to those without. Large consolidations predicted PE with a sensitivity of 77% and a specificity of 71%. The Wells score was remarkably higher in patients with PE compared to those without and predicted PE. By combining the two modalities and using LUS plus a Wells score ≥2 or <2, patients with almost sure/probable PE were compared to patients with possible/unlike PE: PE was predicted with a sensitivity of 100% and a specificity of 80%. Frequently in COVID-19 ARDS patients with PE, large consolidations were detected by LUS. This result should indicate a high-risk for PE in COVID-19 if a Wells score is >2 [119].

## 4. Echocardiography

The evaluation of differential diagnosis for dyspnoea includes also assessment of echocardiography [120]. Transthoracic echocardiography (TTE) is an easy diagnostic tool for early assessment of the cardiac function, but the choice of the echocardiography type (transthoracic-TTE- vs. transoesophageal-TEE) is closely related to the clinical conditions of each patient. Therefore, focused cardiac ultrasound (FoCUS) is part of PoCUS approach. Ventilation represents a key therapy also in emergency settings and in patients on spontaneous breathing and/or non-invasive ventilation. TTE is more feasible, and it can provide an answer for most clinical inquiries.

### 4.1. Left Heart Evaluation

**Figure 3:**
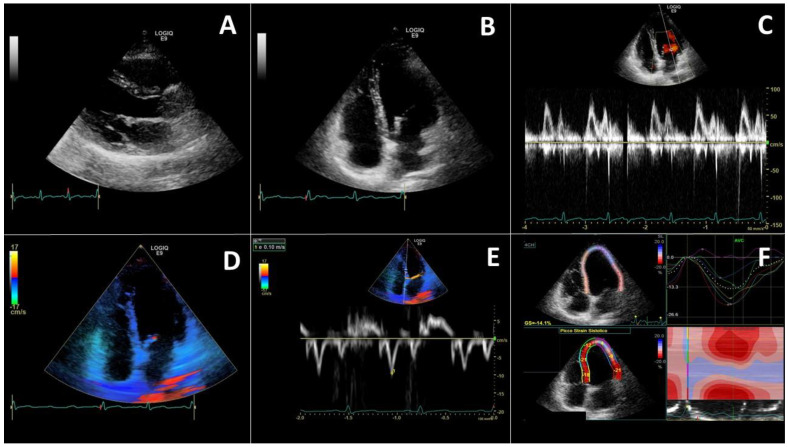
Left heart evaluation needs a complete echocardiography evaluating parasternal long-axis (**A**) and apical 4 chamber (**B**) views. Using pulsed wave Doppler trans-mitral flow is evaluated (**C**). The apical 4 chamber view allows to perform tissue Doppler imaging analysis (**D**), able to make a doppler spectral analysis of the myocardial contraction velocity wave (**E**). A complete evaluation also includes the Speckle tracking evaluation to obtain the longitudinal strain (**F**). In particular the shown image was obtained in a 28-year old girl with a COVID-19 myocarditis.

Global cardiac function might decline in patients with viral infections. A subcostal approach or an apical four-chamber view is useful for a global view of the heart mechanics is obtained through (Figure 3). Through the use of these windows, the diameters of the heart chambers and motion abnormalities are rapidly assessed [117]. The LV function, is an important assessment in a patient presenting shortness of breath, chest pain or sudden drop in arterial blood pressure, aiming at facilitating fast and accurate clinical decision making and therapies [121].

If inotropic drugs are administered, it should be done with caution and a clinically interpreted LV ejection fraction should be measured. Valvular (mitral and aorta) diseases should always be assessed, in particular at first echocardiogram. The presence of moderate-to-severe valvular disease may affect the course and severity of the lung disease and, obviously, therapies (especially fluid administration) [122].

In a prospective international survey, cardiac abnormalities were described in COVID-19 patients [123]. Out of 1216 patients, about 55% had abnormal echocardiogram, in most cases related to LV abnormalities (39%) due to new myocardial infarction (3%), myocarditis (3%) and TCM cardiomyopathy (2%). A severe cardiac disease was described in about 15% patients. Those findings were reported in both with and without a pre-existing cardiac disease, but only in the latter case echocardiographic abnormalities are closely related to the severity of COVID-19 symptoms [123].

In another single-centre study with TTE, 90 patients hospitalized for COVID-19 showed both severe and non-severe [124] echocardiographic features. Among the severe group patients, the right ventricle (RV) and the LV diameters were larger, while LV ejection fraction (LVEF) was decreased, and more frequent pericardial effusions were seen [124].

However, these alterations may not be permanent. In fact, in a short-term follow-up cross-sectional study, neither abnormality was identified in the heart of COVID-19 survivors, nor cardiac differences were detected between patients with different severity of illness, suggesting that patients who recover from COVID-19 do not have considerable cardiac sequelae [125].

Reports on the use of echocardiography in ICU are increasing. The most common reliefs in patients with severe COVID-19 illness are: pulmonary artery hypertension, pericardial effusion, segmental wall motion abnormality and reduced ejection fraction [126]. Other commonly reported echocardiographic findings are: diffuse myocardial wall motion abnormality, LV enlargement and dysfunction, hyperdynamic LV function, TCM, signs of increase in RA pressure including increased IVC diameter and decreased IVC collapsibility [127]. Critical care chest ultrasonography has been used to obtain real time information, enable decision-making in COVID-19 patients. It is useful to provide hemodynamic evaluation as well as cardiac and respiratory function monitoring at patient’s admission and during hospitalization. 

A pragmatic FoCUS based strategy seems the most reasonable approach. Ultrasonography is an adjunct to the physical examination at the point of care to recognize typical ultrasonography signs that suggest a narrow list of potential diagnoses in specific clinical settings. Numerous data support the fact that non-cardiology trained users using small ultrasonography devices can assess heart change elevation more accurately than the physical examination. In addition, FoCUS-trained providers may have skills to perform ultrasonography imaging of body systems outside the heart to supplement their cardiac evaluation [128].

As in emergency, and in ICU, LUS should be combined with FoCUS for a better evaluation of patients with respiratory failure. LUS helps clinical evaluation due its increased power in bedside detection of pulmonary pathologies, monitoring the progression of mechanical ventilation, and detecting complications such as pleural effusion, pneumothorax and atelectasis [112]. Moreover, TTE is a first-line imaging modality in heart assessment, and it is an indispensable bedside tool, allowing non-invasive quantification of cardiac performance in patients hospitalized in isolated wards. It also helps in titration of hemodynamic support, institution and adjustment of disease-specific therapies and optimization of treatment. Guarracino et al. [129] discussed the importance of a comprehensive US approach to the patients’ care. They suggest to perform always simultaneously cardiac, vascular and lung ultrasound in ventilated patients, in order to gain a more comprehensive understanding of the relationship between lung and potential heart and vascular abnormalities [129].

### 4.2. Right Heart Evaluation

Right heart (RH) failure or dysfunction can occur in pre-existing pathologies such as obstructive sleep apnoea, chronic obstructive pulmonary disease, pulmonary hypertension or acute onset of new disease in the critically ill patients [117,121,130,131]. In addition, ventilation can be an induce RH decompensation. Critically ill patients have higher risk of developing pulmonary embolism (PE), and current data suggest the high prevalence of PE in SARS-CoV-2 infections [18].

Cardiac ultrasound might explore RV enlargement. In such patients, the causes are PE, increased pulmonary vascular resistance due to hypoxia or to aggressive non-invasive ventilation (NIV) [116,130,132]. TTE is not the gold standard to diagnose PE, but in the pandemic setting it might be a safe tool for diagnosis, allowing fast treatment [116]. In a patient with PE, it may be found RV hypokinesis with paradoxal septal movement and even akinesia of the RV mid free wall with normal motion of the apex [116,121,132]. In case of massive PE, the LV may result underfilled and hyperdynamic. RV to LV end-diastolic ratio higher than one should guide towards RV failure [116].

RV is directly and indirectly involved in COVID-19 disease course. Therefore, RV echocardiographic assessment has a pivotal role in the understanding of disease status and in monitoring the progression. 

**Figure 4:**
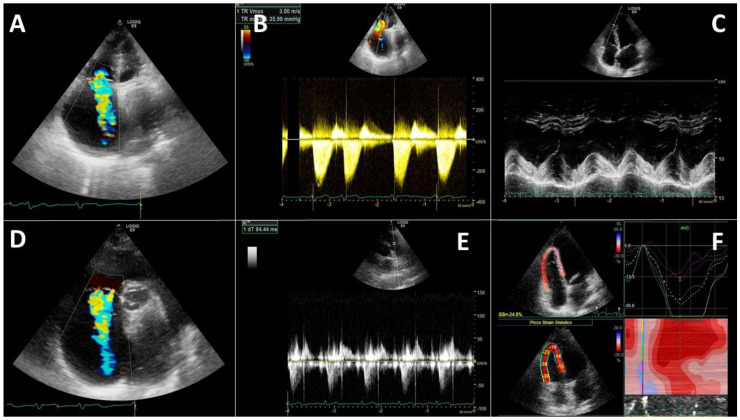
Right heart evaluation needs an apical 4 chamber view (**A**) useful to detect the tricuspid regurgitation velocity (**B**) and the tricuspid annular plane excursion (TAPSE) (**C**). The parasternal short-axis view (**D**) allows another possible evaluation of tricuspid regurgitation as well as the pulmonary artery analysis (**E**). A right ventricle longitudinal strain (**F**) is possible in advanced imaging from an apical 4 chamber view.

The RV direct involvement often recurs. Interstitial pneumonia and pulmonary hypoxic vasoconstriction are two main causes of RV afterload increase. Mechanical ventilation (both non-invasive and invasive) influences heart–lung interaction and affects RV dimension (Figure 4).

RV size can be easily assessed visually by the “eyeball” method in the short-axis and four chamber views. However, it is advisable to perform a quantitative evaluation the RV/LV ratio end-diastolic area [133]. In 39% of COVID-19 patients has been found the RV dilatation/dysfunction, whereas all patients who clinically deteriorated (20%) showed the RV function deterioration [116].

RV wall hypertrophy is defined by diastolic thickness >5 mm. Whenever this finding is detected in Emergency or ICU, it may indicate a chronic RV overload as a consequence of a previously unknown lung disease such as a chronic obstructive pulmonary disease. It has to be considered that RV hypertrophy may be caused by the mechanical ventilation itself in long standing ventilated patients, since the RV is able to thicken in response to increased intrathoracic pressure [134].

Several factors may contribute to the development of increased systolic arterial pressures in COVID-19 patients. Hypoxic pulmonary vasoconstriction is probably the main factor [135]. Secondly, lung disease contributes to alterations in pulmonary circulation, similarly to the role of PEs/thrombosis. Finally, ventilations are known to affect RV afterload [136,137]. The sum of these factors induces an augmented afterload the RV has to face. Therefore, it seems advisable to monitor pulmonary arterial pressure (PAP) using TTE in these patients to obtain an early detection of RV dilatation/dysfunction.

In a retrospective study on 112 COVID-19 patients with mild disease, Deng et al. [126] reported an increased pulmonary hypertension (PH) in 13% of patients. On the contrary, in 28 patients affected by severe disease [138] the systolic PAP was increased in all patients on admission but the authors described a significant decreased during hospitalization. In patients with mild disease, those with PH had a worsening in lung involvement [116,135], while in severe patients shorter pulmonary accelerating time was detected, suggesting increased RV afterload [135].

In non-ventilated patients, Inferior vena cava (IVC) diameter and its collapse is a good estimation of RA pressure. IVC diameter <2.1 cm that collapses >50% during sniff has to be considered indicative for normal RA pressure (3 mmHg), while higher RA pressure (about 15 mmHg) is suggested by an IVC diameter >2.1 cm that collapses <50%. In patients with RV dysfunction, the IVC collapsibility should be interpreted with caution, considering also its trend and central venous pressure [133]. 

An index of RV systolic function is tricuspid annular plane systolic excursion (TAPSE). (Figure 3). TAPSE <15 mm indicates a RV dysfunction, and it is associated with poor prognosis in critically ill patients.

RV overload has also a prognostic role. In a prospective study on 94 consecutive patients, coupling RV function to the pulmonary circulation was evaluated as TAPSE to PAPs ratio [132]. The authors found that in non-survivors, PAPs was increased while TAPSE decreased, and the TAPSE/PAPs ratio was lower than in the survivors. The latter parameter results also as the only independent predictor of mortality (cut-off 0.635 mm/mmHg) next to P/F value at univariate/multivariable analysis [132].

### 4.3. Cardiovascular Ultrasound Evaluation in Different COVID-19 Phenotypes

Direct cardiac involvement may present as different entities. The most described alterations are the detection of localized wall motion abnormalities, as a global ventricular depression in case of myocardial infarction with ST-elevation, or as dilated cardiomyopathy, as a severe decrease in LV systolic function and pericardial effusion in case of viral myocarditis [139]. In COVID-19 pneumonia, it may also occur an indirect involvement of the heart, that differs between two pulmonary phenotypes [140]: the L phenotype, which has a preserved compliance, and the H phenotype, with high pulmonary elastance. Heart abnormalities related to the previous phenotypes should be identified. In the L phenotype, during the setting of protective ventilation, the pressure to deliver tidal volume is lower, thus a less severe impairment of RH would be expected. In this kind of dyspnoeic patients, either spontaneously breathing or on non-invasive respiratory support, echocardiography may reveal ventricular interdependence due to the increased respiratory effort. Dyspnoea causes considerable pleural negative pressure. The intrathoracic change in pressure induces a diastolic ventricular septal shift, leading to LV hypodiastole and reduced stroke volume. In contrast, repercussion on the RH is more probable in the H phenotype because of positive pressure of mechanical ventilation. Hypoxic vasoconstriction of pulmonary circulation and superimposed pulmonary thromboembolic events may further precipitate the abovementioned effects. Echocardiography reveals cardiac damages directly related to ventilation. In particular, the previously reported alterations secondary to mechanical ventilation are found, with regard to RV involvement, leading to ventricular dilation, reduced systolic RH function, tricuspid insufficiency and possible secondary left heart compression resulting in reduction of LV stroke volume [129]. A ventilator-induced heart dysfunction in patient with no previous cardiac dysfunction has been described [141]. Positive inspiratory pressure of mechanical ventilation has less influence on LV function, which needs to be assessed because it can be directly altered by RH dysfunction. Systemic arterial hypertension is reported in about 50% of COVID-19 pneumonia patients, therefore diastolic function should always be closely evaluated. Likewise, specific attention should be addressed to the diastolic profile of COVID-19 patients, whose lung ultrasound reveals a B pattern, particularly when it is associated with reduced LV function. TTE is useful to guide tailored therapeutic strategy because it helps clinicians in identifying high risk for ventilator weaning failure. Finally, when circulation support with extracorporeal membrane oxygenation (ECMO) is needed, the basis of concomitant cardiogenic cause may be explored using both TTE and TEE, in order to decide the device selection (venovenous vs. venoarterial), to assist device placement (cannulation) and monitoring cardiac function as well as device-related complications [56].

García-Cruz et al. [142] confirmed the important use of ultrasounds, and introduced the “ORACLE” protocol, to enable a quick image acquisition at the patient’s bedside in approximately 20 min, while the image analysis was performed outside the patient’s room to reduce the operators’ risk of infection. The protocol included evaluation of left (O) and right (R) ventricular function, valves (A), pericardial effusion (C), stratification of the severity of pulmonary affection (L) based on the LUS score next to the evaluation of pulmonary haemodynamics, regional wall motion, cardiac output, diastolic function and filling pressures, and fluid responsiveness with IVC distensibility index (E). They pointed out how the application of the ORACLE protocol in 82 patients with severe disease had an impact on the patient’s management. In fact, the protocol has been useful to obtain fluid infusion and preload optimization in patients with fluid responsiveness, to initiate inotropes in those with RV dysfunction and TCM, to avoid fluid overload in patients with ARDS, helping in pericardial drainage when cardiac tamponade is detected [142].

## 5. Ultrasound for Deep Vein Thrombosis Evaluation

Compressive ultrasound (CUS) for DVT [143] should be used for patients hospitalized for COVID-19 either with clinical symptoms of deep vein thrombosis (DVT). When there are not clinical symptoms elevated D-dimers levels or DVT clinical probability (Wells score > 2) are useful to guide clinicians.

The 4-point CUS has been widely used both in USA and Europe, as a non-invasive method of compression enabling emergency physician in screening for suspected DVT. Analysing the absence of proximal venous incompressibility at the four points (femoral and popliteal, right and left) [122] it is possible to exclude DVT. Total or partial vein incompressibility is indirect sign of DVT, and it is the only parameter required for diagnosis. The 4-point ultrasound is simple, reliable (with a sensitivity and specificity of 90–100%), safe, available, inexpensive, and rapid (between 3 and 5 min), and reduces the potential exposure to the virus limiting the risk of contamination.

In a prospective observational study, consecutive COVID-19 patients with a diagnosis of PE were ultrasound screened for DVT in the lower extremities [143]. DVT was diagnosed in the emergency department in about 7% of patients who had central and bilateral PE. However, patients without DVT had higher median of D-dimer levels, suggesting that PE should mainly result as local thrombo-inflammatory syndrome and not a real thromboembolic event.

Accumulating evidence suggests that despite the use of anticoagulant therapy, there is a high incidence of thromboembolic events leading to the hyperinflammatory state especially in ICU patients [144]. It is worth noticing that most ICU patients receive a central venous catheter (CVC) due to their need for infusion of vasoactive agents or haemodialysis. However, many COVID-19 patients are often affected by catheter-related thrombosis (CRT). This is considered a serious complication due its correlation to PE, increase the risk of infections or long-term central venous stenosis. As a consequence, catheter dysfunction is associated with considerable healthcare costs [145]. Since COVID-19 coagulopathy causes an overall hypercoagulability rather than a local pulmonary prothrombotic state, CRT is frequent in critically ill patients who have an indwelling CVC. In a multicentre case–control study [146], the hypothesis that COVID-19 predisposes to CRT in critically ill patients was evaluated. The study population consisted of 82 critically ill adult patients admitted to the ICU who had an indwelling or recently removed (≤48 h) CVC in the internal jugular, subclavian or femoral vein. At time of CVC insertion, standard dosage thromboprophylaxis was doubled and based on the body weight. Multiple certified operators performed ultrasound compressive examination of the CVC entry vein. This entry vein was scanned by compression every 2 cm, and B-mode ultrasound was used to assess residual flow. As diagnostic for CRT, it was considered if the vein failed to collapse at any point or an echogenic thrombus or intraluminal filling defect were detected. The control group consisted of patients that were ruled out for CRT. Compared to controls, the COVID-19 exposure results in a crude OR of 7.2 for CRT rising to 18.3 if adjusted for anticoagulant usage and catheter indwelling time [146]. There preliminary results were tested also in large cohort Italian study, confirming that COVID-19 had a statistical higher rate of CRT as compared to disease free patients despite prophylactic heparin therapy was performed [147]. Therefore, in agreement with previous researches investigating thrombotic complications in ICU patients, COVID-19 highly predisposes critically ill patients to CRT [65].

Moreover, in patients requiring venovenous ECMO, the occurrence of VTE has been studied only using ultrasonography. Parzy et al. [148] through CT scan imaging report a 100% occurrence of VTE in 14 critically ill patients supported by venovenous ECMO for ARDS. These events occur despite a high target and close monitoring of anticoagulation. All this evidence demonstrates that in patients that underwent ECMO during SARS-CoV-2 infection there is an interplay leading to coagulation and thrombosis. In the light of the above, clinicians must be aware of the complications in critically ill patients due to the required attention for thrombosis prevention and diagnosis.

## 6. Chest Computerized Tomography in COVID-19

Computed tomography (CT) is highly sensitive for diagnosis of patients with COVID-19, but it might not always be available. Several studies underlined the importance of imaging for COVID-19 diagnosing [149]. The typical COVID-19 CT feature is ground-glass opacities (GGO) or mixed GGO, consolidation and vascular enlargement. Lesions are generally lower-lung predominant and usually display a peripheral distribution and bilateral involvement [149].

Next to GGO, the usually evaluated parameters in chest CT scans are: increased opacity of the lung, with preservation of bronchial and vascular margins, consolidation, crazy-paving pattern, cavity and bronchial dilatation [150,151]. Vascular involvement is indicated by vessel dilatation, an increased vessel diameter within or near opacifications, larger compared to vessels in healthy lung tissue [150].

Thromboembolic complications with or without the presence of DVT are frequent and underestimated events. The diagnosis of PE mainly relies on CT scanning, but ultrasound is a diagnostic alternative for special clinical settings [120]. Performed as contrast-enhanced CT pulmonary angiography, it can detect or rule-out the PE: in fact, emerging evidence describes an increased hypercoagulability in COVID-19 patients. 

The overall prevalence of acute PE diagnosed through CT ranged between 14–30% [65,152,153]. Noteworthy, despite prophylactic or therapeutic anticoagulation, the rate of PE in severe patients admitted to hospital ranged between 25–26%, supporting the hypothesis of a correlation between the severity of the disease and the prothrombotic phenotype of severely ill patients [144,154]. On the basis of thrombi’s location, considering the most proximal pulmonary arterial branch involved, four types of PE (subsegmental, segmental, lobar and central) were defined [143,155]. Of note, segmental is the most frequent distribution of pulmonary emboli (51%) followed by lobar (31%) and central (13%) [156]. CT pulmonary angiography (CTPA) can assess not only the presence of pulmonary embolus but also the severity of the embolus as well as heart function and strain on the RV.

**Figure 5:**
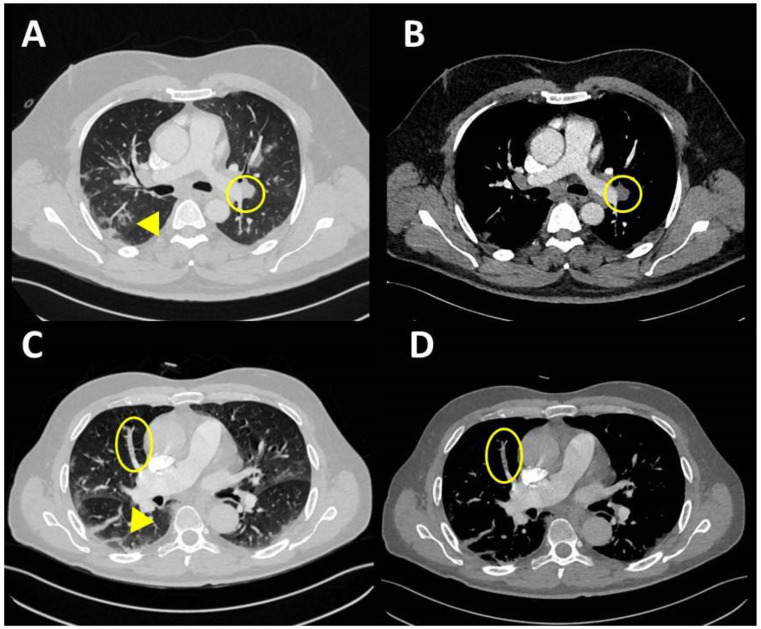
CT scan is able to identify both gross pulmonary embolism (**A**,**B**) and tinier obstructions in subsegmental artery (**C**,**D**). Embolism (Circles) is not related to site of pneumonia but to endotheliitis and not to pulmonary inflammation (Triangles).

CTPA is becoming the standard of care for the evaluation of patients with suspected PE. In acute PE the diagnostic criteria include: possibly enlarged artery compared with adjacent patent vessels and/or arterial occlusion due to a large filling defect highlighted by failure in enhancing the entire lumen [144,153] (Figure 5).

It has been demonstrated that acute PE is statistically significant associated to peripheral wedge-shaped areas of hyper-attenuation that may represent infarcts, along with linear bands. On the contrary, chronic PE can manifest as complete occlusive disease in vessels smaller than adjacent patent ones. Other CTPA findings in chronic PE include webs or flaps, recanalization and partial filling defects [151,157,158].

CT is currently deemed as the most sensitive imaging tool when COVID-19 is suspected, being capable to detect specific and highly suggestive signs: ground-glass opacities with or without consolidation in the lung periphery. Cardiac involvement is being frequently reported in chest CT. This procedure is useful to selected patients with elevated cardiac biomarkers, in case of inconclusive echocardiography and signs and symptoms of an acute coronary syndrome to rule out coronary artery disease. It is an important tool for diagnosing stenosis, valve dysfunction, coronary dissection and intra-cardiac device dysfunction. In various reports, pericardial effusion [34] and evidence of myocarditis, including increased wall thickness, myocardial oedema and hypokinesia, have been described [159]. 

## 7. Radiography in COVID-19 Evaluation

The overlap of infectious symptoms and classic symptoms of cardiac syndromes presents a diagnostic challenge; therefore, awareness and vigilant surveillance for possible cardiovascular sequelae is critical. In this context, cardiovascular imaging offers an important instrument to facilitate the diagnosis of clinically suspected conditions. Various imaging modalities contribute to diagnostic evaluation, management and prognosis such as chest x-ray, echocardiography, chest computed tomography (CT), magnetic cardio-resonance and coronary angiography. 

Radiology is an important complement to clinical and epidemiological features, both in adult and paediatric population. Chest x-ray is frequently requested in patients with acute pulmonary symptoms admitted to the emergency department, as well as in the ICU, because it is inexpensive, suitable to be placed by the patient’s bed and has low radiation exposure. The most common findings refer to lung abnormalities while pleural effusion and altered cardiomediastinal contour are infrequent. Cardiac findings on chest x-ray are restricted to enlargement of cardiac silhouette and the presence of pulmonary oedema. 

## 8. COVID-19 Specificity Advanced Imaging

Advanced cardiac imaging may play a role in discriminating the broad spectrum of differential diagnoses. The easiest tools are represented by the advanced imaging techniques in echocardiography. Among them, the most important one is the myocardial strain evaluation using the speckle-tracking analysis. Similarly, other useful tools are the cardiac magnetic resonance (CMR) and the positron-emission tomography (PET) that, however, are both more difficult to use in acute patients. Finally, there is no specific advanced imaging useful to differentiate or help in arrhythmias’ management. Nevertheless, being the latter issue an essential prospective, additional studies are needed [160] to further investigate this topic.

### 8.1. Echocardiography Longitudinal Strain

TTE may be empowered by advanced imaging techniques. The longitudinal strain (LS) measured by two-dimensional speckle-tracking echocardiography (2D-STE). It is a recent method able to perform a more accurate and sensitive indicator of cardiac function in a variety of cardiovascular diseases [161,162] and a prognostic tool in different clinical settings [163,164]. RV LS (RVLS) is an evolution of the technique used in COVID-19 patients.

Xie et al. [165] investigated biventricular LS in COVID-19 patients as prognostic tool. They found that patients with cardiac injury had higher levels of inflammatory and coagulopathy markers, more mechanical ventilation therapies, higher incidence of complications, and higher mortality. Heart involved patients were characterized by decreased of both LV and RV strain. These results were correlated with higher biomarkers of cardiac injury and inflammation as well as by the presence of pericardial effusion [165,166]. Moreover, patients who died were afflicted by impaired LS, an independent predictor of mortality in a Cox analysis. Therefore, at 3-month follow-up visit after discharge in survivors, a significant improvement was observed in both right and left LS [165]. However, RVLS worsened in patients who experienced hospitalization due to their clinical conditions, especially if they had severe pneumonia [166].

Furthermore, RVLS is useful to evaluate patients who received ventilation. Abnormal LS was found in about 66% of patients ventilated in ICU, and it was associated with higher lung compliance, lower airway plateau pressures, lower tidal volume ventilation and reduced LV function [167]. RV LS is not related to abnormal lung mechanics or ventilatory pressures [167]. A recent systematic review on LS both of LV and RV in COVID-19 patients showed that lower LV-GLS and RV-LS were associated with poor outcome [168]. Authors evaluated clinical trials that analysed as outcome a composite of mortality and severe COVID-19. Their meta-analysis included 7 studies and 612 patients. They found that each 1% decrease in LV-GLS was associated with 1.4 increased risk of poor outcome, while each 1% decrease in RV-LS was associated with 1.3 increased risk of poor outcome [168].

### 8.2. Heart Computerized Tomography

Computerized tomography (CT) emerged as one of the primary imaging modalities in the COVID era. CT can be used to evaluate chest pain, LV dysfunction, new onset of heart failure or cardiomyopathy, and evaluation of patients with possible angina and new arrhythmias [6]. Cardiac CT also allows the have a comprehensive assessment of pulmonary parenchyma and vessels as well as an evaluation of coronary arteries. Cardiac CT use become the preferred tool in acute atrial arrhythmias to evaluate LA before to cardioversion, to evaluate of LA appendage closure and during atrial fibrillation ablation [6]. Endocarditis evaluation changed to incorporate multiphase cardiac CT instead of the TEE for patients with low-risk echocardiography findings who presents ongoing clinical suspicion because a persistent bacteraemia. For patients requiring surgical intervention for endocarditis, cardiac CT is preferred to both pre-operative TEE and cardiac catheterization which were routinely deferred when possible. In fact, cardiac CT is able to perform an evaluation of peri-valvular complications and coronary anatomy in patients without known coronary disease. However, it is limited to expert centres and it is not routinely used.

### 8.3. Cardiovascular Magnetic Resonance

In COVID-19 patients is not clear the role of cardiovascular magnetic resonance (CMR). Advances in multi-parametric CMR now allow having a detailed tissue characterization including scar, diffuse fibrosis, oedema and quantitative ischaemia assessment. During the COVID-19 pandemic, CMR has not been widely considered, as for the necessary limitations due to the strain put upon the healthcare system [169]. Accepted diagnostic indications for CMR should be considered appropriate in COVID 19 patients, but only if it is clinically necessary and after other suited imaging techniques were assessed. Special attention should be given to the use of gadolinium enhanced CMR in patients with COVID-19. Kidney function is often decreased and might contradict a clinically urgent CMR scan. In these patients, the importance of enhanced adrenergic stimulation, systemic inflammation and renal failure in more advanced cases should be recognised. Details of CMR are rather limited but it is useful to provide a diagnosis in patient with elevated troponin from unclear aetiology [170]. Whenever feasible, CMR can allow a non-invasive diagnosis of clinically suspected myocarditis, while a definite diagnosis and proof of SARS-CoV-2 infection and inflammation would require endomyocardial biopsy. The suspicion of acute myocarditis might be one indication for a CMR, especially if typical symptoms, elevated troponins, ventricular dysfunction and/or severe arrhythmias cannot be explained by other diagnostics and imaging methods.

Esposito et al. reported a series of eight patients with elevated troponin and electrocardiography alterations whose CMR findings fulfilled the 2018 Lake Louise Criteria for the diagnosis of myocarditis [171]. Despite that all patients included had no remarkable previous history of cardiovascular disease, CMR showed diffuse intense myocardial oedema, increased T1 and T2 mapping and a mild pericardial effusion in 75% of them [160]. However, other reports suggest that CMR patterns are heterogeneous but similar to other typical form of active myocardial inflammation characterized by diffuse oedema. Late gadolinium enhancement (LGE) seems to be less-frequently observed in these patients [171], indicating a myocyte necrosis is limited at acute phase [160]. In fact, LGE has a non-ischemic pattern and it is predominantly located in the inferior and inferior-lateral segments [172].

CMR may be helpful to evaluate the presence of a myocardial injury. In this setting, patients may present a decreased LV ejection fraction, increased LV volumes, and raised native T1 and T2. Wang et al. [173] confirmed these results in a small cohort of patients evaluated with CMR three months after recovery: LGE was found in 30% of them. These patients had significantly decreased LV peak global circumferential strain (GCS), RV peak both GCS and GLS as compared to non-LGE patients. In contrast, no difference was found between healthy controls and non-LGE patients. Lesions were located in the mid myocardium and/or sub-epicardium with a scattered distribution [173]. In the so far largest prospective observational cohort study, Puntmann et al. [174] has described abnormal CMR findings, including raised myocardial native T1, raised myocardial native T2, myocardial LGE or pericardial enhancement. A small but significant difference between patients who recovered at home vs. hospital for native T1 but not for native T2 mapping was described. Native T1 and T2 mapping is significantly correlated to high-sensitivity troponin T and it is independent of pre-existing conditions, severity and overall course of the acute illness [174].

### 8.4. Other Advanced Tools in COVID-19 Imaging

Cardiac evaluation of metabolic pattern should be assessed using [18F]-2-Fluoro-2-deoxy-D-glucose (FDG) positron emission tomography (PET) [175]. It is a sensitive and quantitative technique to detect inflammatory process. Few cases are reported on the use of FDG-PET in COVID-19 patients [176,177,178]. Recently, Dietz et al. [179] using FDG-PET assessed the inflammatory status at the presumed peak of the inflammatory phase in non-critically ill patients. Next to lung inflammation, all patients demonstrated increased mediastinal lymph nodes glucose uptake. The worthy finding is that they described also a myocardial up-take related to SARS-CoV-2 infection [179]. Despite the fascinating perspectives, larger forthcoming studies are needed to evaluate FDG-PET utility for COVID-19 myocardial damage.

An additional type of imaging is the coronary angiography (CAG): it is the gold standard to evaluate the coronary artery lumen, and it is recommended only in patients with ST-segment elevation on EKG or with new left bundle-branch block. In clinically suspected STEMI, coronary angiography should be performed whenever possible, since COVID-19 can trigger acute coronary syndromes. At the same time, alternative causes of troponin elevation must be investigated, if STEMI is excluded.

Artificial intelligence (AI) is a brand new tool rising in helping the daily medical practice and clinical imaging [180]. The use of AI has been also proposed for COVID-19 [181,182] treatment. In CT, CMR and ultrasound modalities, AI has been applied for data retrieval, segmentation of medical organs and diagnosis for COVID-19 [183]. However, AI is far from being routinely used in daily practice or in acute settings.

## 9. Safety Concerns

**Figure 6:**
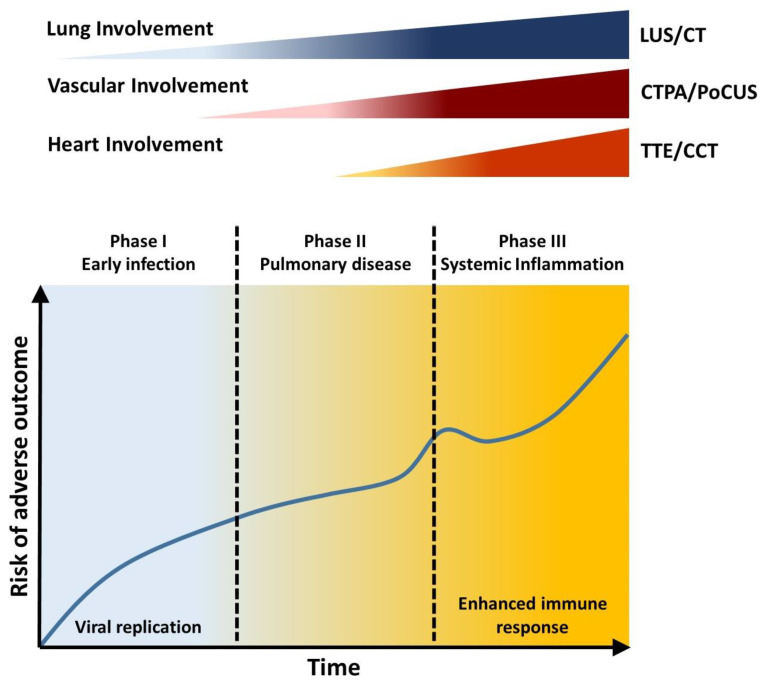
Viral replication and host immune response cooperate in disease progression. Through the three stages, different chest imaging modalities are useful to study the cardiovascular involvement. Transthoracic echocardiography (TTE) can identify left ventricle impairment as well as increased pulmonary hypertension and right ventricular dysfunction. Viral infection or to systemic inflammation can induce cardiovascular complications at different stages of the disease, inducing an increased risk for adverse outcome. To assess cardiovascular involvement specific multimodality imaging is required. CT: computed tomography; CTPA: computed tomography pulmonary arteriography; LUS: lung ultrasound; TTE: transthoracic echocardiography.

In the COVID-19 era, the safety of healthcare providers and patients remains a universal priority regardless of the imaging modality used. Recommendations on imaging in COVID-19 emphasized the importance of proper PPE equipment for healthcare. They also recommend to limit testing to the ones which have an impact on patient health as well as clinical management [184,185,186,187]. In ventilated patients, especially those ventilated mechanically, TEE is able to overcome technical problems with acoustic views. However, to limit the virus spread exposure, TEE is recommended to selected patients. Both the European Society of Cardiology [187] and American Society of Echocardiography [185] suggest limiting its use. In fact, TEE has inherent risks and limitations related to manpower and infection control. Airborne precautions are required during a TEE for suspected and confirmed cases, due to the increased risk for aerosolization [185,187]. The value of TEE in COVID-19 pandemic is seen in familiar domains, for instance patients whose adequate transthoracic echocardiographic windows cannot be generated. Similarly, frequent scenarios in ICU care of COVID-19 need to be addressed using TEE, such as hemodynamic instability during prone ventilation, serial evaluations of the lungs, during cardiac arrest resuscitation, and to guide venovenous ECMO cannulation [188]. However, comparison of possible advantages and disadvantages in different clinical settings (Table 1, Figure 6) may help to choose the specific test and reduce the infection exposure.

## 10. Conclusions

During the COVID-19 pandemic, healthcare systems and health workers are making every effort to ensure the best treatments to each patient. In view of resources optimization, imaging can effectively support the assessment and prognostic evaluation of critically ill patients. The different techniques are able to provide different information. Ultrasound is the most reliable and easy to use in emergency settings and in the ICU as a first approach. It is also an amazing tool for frequent instrumental follow-ups. However, for many other diseases, there is the need for an increased diagnostic power, that only more complex machines are able to ensure. For this reason, a combination of different methods is the best possible way to perform adequate patient care and reduce operators’ exposure to infection. Additional prospective studies are needed to develop an effective method which combines the different tools.

## Figures and Tables

**Table 1:** Characteristics of each imaging modalities for care of COVID-19 patient.

Imaging Modality	Emergency		Intensive Care		COVID-19 Findings
	Advantages	Disadvantages	Advantages	Disadvantages	
Point-of-care ultrasound	Rapid Performed bedside No radiation Low cost Minimal equipment	Infectious exposure to provider Image quality compromised by patient habitus or ventilation More limited functionality compared to echocardiography	Rapid Performed bedside No radiation Low cost Minimal equipment	Infectious exposure to provider Image quality compromised by patient habitus or ventilation More limited functionality compared to echocardiography	Basic LV and RV structural and functional abnormalities Pericardial effusion Pleural effusion B lines (may indicate interstitial oedema on lung ultrasound)
Echocardiography	Performed bedside No radiation Low cost	Sonographer infectious exposure Image quality often compromised by patient habitus or ventilation	Performed bedside No radiation Low cost	Sonographer infectious exposure Image quality often compromised by patient habitus or ventilation	RV dilation and dysfunction LV systolic and diastolic dysfunction Wall motion abnormalities Stress cardiomyopathy Pulmonary hypertension Reduced LV and RV strain Pericardial effusion Elevated filling pressures
CT	Rapid High resolution Moderate cost Some tissue characterization	Radiation Risks of iodine contrast Not bedside Difficult disinfection	Rapid High resolution Moderate cost Some tissue characterization	Radiation Risks of iodine contrast Not bedside Difficult disinfection	Pulmonary embolism Cardiomegaly Chamber size Intracardiac thrombus Pericardial effusion
CMR		No indication in emergency	High resolution Functional imaging Superior tissue characterization No radiation	Expensive Not bedside Time-consuming Frequent patient intolerance and incompatibilities Difficult disinfection	Ischemic vs. non-ischaemic injury Stress cardiomyopathy Myocarditis Pericarditis Chamber enlargement Strain abnormalities
Nuclear imaging	Inflammation localization	Low resolution Not bedside Time-consuming Radiation exposure Difficult disinfection Limited indication in emergency	Inflammation localization	Low resolution Not bedside Time-consuming Radiation exposure Difficult disinfection	Valvular inflammation in endocarditis (FDG-PET alternative to TEE) Myocardial inflammation in myocarditis
